# PLAU promotes growth and attenuates cisplatin chemosensitivity in *ARID1A*-depleted non-small cell lung cancer through interaction with TM4SF1

**DOI:** 10.1186/s13062-024-00452-7

**Published:** 2024-01-17

**Authors:** Yuanliang Zheng, Lixiang Zhang, Kangliang Zhang, Shenghao Wu, Chichao Wang, Risheng Huang, Hongli Liao

**Affiliations:** 1 Department of Thoracic Surgery, The Dingli Clinical College of Wenzhou Medical University, Wenzhou Central Hospital, The Second Affiliated Hospital of Shanghai University, Wenzhou, China; 2 Department of Central Lab, The Dingli Clinical College of Wenzhou Medical University, Wenzhou Central Hospital, The Second Affiliated Hospital of Shanghai University, Wenzhou, China; 3 Department of Hematology and Chemotherapy, The Dingli Clinical College of Wenzhou Medical University, Wenzhou Central Hospital, The Second Affiliated Hospital of Shanghai University, Wenzhou, China; 4 Department of Pathology, The Dingli Clinical College of Wenzhou Medical University, Wenzhou Central Hospital, The Second Affiliated Hospital of Shanghai University, Wenzhou, China

**Keywords:** ARID1A, Chemoresistance, Growth, Lung cancer, PLAU

## Abstract

Loss of *ARID1A*, a subunit of the SWI/SNF chromatin remodeling complex, contributes to malignant progression in multiple cancers including non-small cell lung cancer (NSCLC). In the search for key genes mediating the aggressive phenotype caused by *ARID1A* loss, we analyzed 3 Gene Expression Omnibus (GEO) datasets that contain RNA sequencing data from *ARID1A*-depleted cancer cells. PLAU was identified as a common gene that was induced in different cancer cells upon *ARID1A* depletion. Overexpression of PLAU positively modulated NSCLC cell growth, colony formation, cisplatin resistance, and survival under serum deprivation. Moreover, enforced expression of PLAU enhanced tumorigenesis of NSCLC cells in nude mice. Mechanistically, PLAU interacted with TM4SF1 to promote the activation of Akt signaling. TM4SF1-overexpressing NSCLC cells resembled those with PLAU overepxression. Knockdown of TM4SF1 inhibited the growth and survival and increased cisplatin sensitivity in NSCLC cells. The interaction between PLAU and TM4SF1 led to the activation of Akt signaling that endowed *ARID1A*-depleted NSCLC cells with aggressive properties. In addition, treatment with anti-TM4SF1 neutralizing antibody reduced the growth, cisplatin resistance, and tumorigenesis of *ARID1A*-depleted NSCLC cells. Taken together, PLAU serves as a target gene of ARID1A and promotes NSCLC growth, survival, and cisplatin resistance by stabilizing TM4SF1. Targeting TM4SF1 may be a promising therapeutic strategy for *ARID1A*-mutated NSCLC.

**Supplementary Information:**

The online version contains supplementary material available at 10.1186/s13062-024-00452-7.

## Introduction

Lung cancer is the leading cause of cancer-related deaths worldwide, causing approximately 2 million deaths per year [1]. Non-small cell lung cancer (NSCLC), comprising the squamous cell carcinoma, adenocarcinoma, and large cell cancer subtypes, represents over 85% of lung cancers [2]. Dramatic advances have been achieved in systemic therapies against advanced NSCLCs [3]. However, most patients with advanced NSCLCs still develop resistance, although they have an initial therapeutic response [4, 5]. Hence, it is important to ascertain the key molecular mechanism(s) underlying NSCLC progression and drug resistance.

The AT-rich interaction domain 1 A (ARID1A) protein is a subunit of the SWItch Sucrose non-fermentable (SWI/SNF) chromatin remodeling complex, which regulates the chromatin structure and enables transcription factor binding [6]. The SWI/SNF complex plays a widespread role in the epigenetic regulation of gene expression [7]. Among the genes encoding the SWI/SNF complex subunits, *ARID1A* gene is the most frequently mutated in human cancers, with an overall mutation rate of ~ 6% [8]. *ARID1A* serves as a tumor suppressor gene. Its mutation leads to loss of function, thus contributing to tumor development and progression [9, 10]. Fukunaga et al. [11] reported that loss of *Arid1a* and *Pten* accelerates malignant transformation of pancreatic ductal cells. Luo et al. [12] reported that ablation of ARID1A leads to transcriptional activation of multidrug resistance-associated protein 2 (MRP2), which induces chemoresistance. In esophageal cancer [13] and lung cancer [14], loss of *ARID1A* promotes invasive growth and metastasis. These studies indicate that ARID1A plays a central role in the prevention of malignant disorders.

Plasminogen activator urokinase (PLAU), also named urokinase-type plasminogen activator (uPA), is a serine protease that catalyzes the transformation of plasminogen to plasmin [15]. It regulates tissue remodeling and cell migration. Accumulating evidence has linked PLAU to oncogenesis [16–18]. Chen et al. [16] showed that PLAU promotes the proliferation and epithelial-mesenchymal transition (EMT) in head and neck squamous cell carcinoma, which is associated with enrichment of the genes involved in EMT pathways. Besides as an enzyme, PLAU can also exert its biological effects through the interaction with other proteins and induction of signal transduction. PLAU has been documented to interact with GLIPR1 to activate EGFR signaling, consequently preventing from cigarette smoke-induced inflammatory response and airway damage [19]. PLAU shows the ability to activate NF-κB signaling to drive the development of cholangiocarcinoma [20]. PLAU is upregulated in lung cancer and can promote lung cancer cell invasion [21, 22]. However, the mechanism of PLAU-mediated aggressive phenotype in cancer cells is largely unknown.

Given the frequent mutation of *ARID1A* in various cancers including NSCLC, we speculated that some key target genes might be derepressed upon *ARID1A* loss and contribute to the progression and drug resistance of *ARID1A*-mutated NSCLC. Here, we analyzed 3 publicly available RNA-sequencing datasets that reflect gene expression changes between *ARID1A*-depleted and control cancer cells. We validated *PLAU* as a target gene of ARID1A. The function of PLAU in *ARID1A*-depleted NSCLC cells was investigated. The PLAU-interacting proteins were examined to uncover the mechanism of PLAU action in *ARID1A*-depleted NSCLC.

## Materials and methods

### Bioinformatic analyses

We downloaded RNA sequencing data from 3 Gene Expression Omnibus (GEO) datasets (GSE218822, GSE180468, and GSE132359). Differentially expressed genes (DEGs) between *ARID1A*-mutated and control cancer cells were determined using the online GEO2R tool (https://www.ncbi.nlm.nih.gov/geo/geo2r/). The Kaplan-Meier Plotter tool (https://kmplot.com/analysis/) was applied to analyze the relationship between gene expression data and overall survival of lung squamous cell carcinoma (LUSC) and lung adenocarcinoma (LUAD) patients. The correlations between *ARID1A* and candidate genes were determined using the Encyclopedia of RNA Interactomes (ENCORI) database (https://rnasysu.com/encori/).

### Cell culture and treatment

The NSCLC cell lines A549 and H1299 were purchased from the American Type Culture Collection (ATCC, Rockville, MD, USA). The cells were cultured in Dulbecco’s modified Eagle medium (DMEM) containing 10% fetal bovine serum (FBS; Invitrogen, Carlsbad, CA, USA) in a humidified incubator at 37 °C and 5% CO_2_. In some experiments, cells were treated with 20 µM of MK2206 (a specific Akt inhibitor) for 6 h before further experiments [23]. To block the TM4SF1 activity of cancer cells, the culture medium with anti-TM4SF1 antibody (2 µg/mL; catalog number: MABC1723; Sigma-Aldrich, St. Louis, MO, USA) [24] was used and replenished every 3 days.

### Plasmids and cell transfection

Two independent *ARID1A*-, *PLAU*-, and *TM4SF1*-targeting short hairpin RNAs (shRNAs) were inserted into the pLKO.1 puro vector. The shRNA sequences are listed in Supplementary Table S1. PLAU- and TM4SF1-expressing plasmids were generated by cloning *PLAU* and *TM4SF1* cDNAs into the pcDNA3.1(+) vector. All cell transfections were performed using Lipofectamine 3000 (Invitrogen) as per the manufacturer’s instructions.

### Western blot analysis

Cells were lysed using radioimmunoprecipitation assay buffer (Thermo Fisher Scientific, Waltham, MA, USA) with a protease inhibitor cocktail (Roche, Basel, Switzerland) for 30 min at 4 °C. Protein concentrations were quantitated using a BCA protein assay kit (Thermo Fisher Scientific). Protein samples were separated by sodium dodecyl sulfate-polyacrylamide gel electrophoresis (SDS-PAGE) and transferred onto nitrocellulose membranes. Protein levels were detected using the primary antibodies: anti-ARID1A (#24,414; Cell Signaling Technology, Danvers, MA, USA), anti-PLAU (#15,800; Cell Signaling Technology), anti-phospho-Akt (#4060; Cell Signaling Technology), anti-Akt (#9272; Cell Signaling Technology), anti-GAPDH (#2118; Cell Signaling Technology), and anti-TM4SF1 (PA5-21119; Thermo Fisher Scientific). Horseradish peroxidase-labeled secondary antibodies (Cell Signaling Technology) were then used. Protein bands were visualized by enhanced chemiluminescence.

### Quantitative real-time PCR (qRT-PCR) analysis

Total RNA was isolated using TRIzol reagent (Invitrogen). Complementary DNA was synthesized using the PrimeScript 1st Strand cDNA Synthesis Kit (Takara, Dalian, China). mRNA expression was quantitated by qRT-PCR using SYBR Green PCR Master Mix (Thermo Fisher Scientific). Relative gene expression was normalized to *GAPDH*. Primer sequences used are listed in Supplementary Table S1.

### Chromatin immunoprecipitation (ChIP)

ChIP assay was conducted using an EZ-ChIP kit (Sigma-Aldrich) as per the manufacturer’s instructions. Cells were cross-linked with 1% formaldehyde and lysed in lysis buffer. Chromatin was sonicated to yield DNA fragments of 200–500 bp. DNA fragments were incubated with anti-ARID1A (PA5-85568; Thermo Fisher Scientific) or normal IgG overnight at 4 °C. The immunoprecipitated DNA was extracted and amplified by real-time PCR. The PCR primers are listed in Supplementary Table S1.

### Luciferase reporter assay

The *PLAU* promoter region (-2000 to + 100 bp) was amplified by PCR and cloned to the pGL3-Basic vector (Promega, Madison, WI, USA), upstream of the luciferase gene. ARID1A-depleted and control NSCLC cells were co-transfected with the *PLAU* promoter luciferase reporter construct and *Renilla* luciferase reporter pRL-TK (used as the internal control for transfection efficiency) using Lipofectamine 3000. After 24 h, luciferase activities were measured using the Dual-Luciferase Reporter Assay System (Promega).

### Cell proliferation assay

Cells were plated in 96-well plates (1 × 10^3^ cells/well) and allowed to grow for 5 days. At indicated time points, the cell suspension was added with trypan blue solution (Sigma-Aldrich) and incubated for 5 min. Cells were counted using a hemocytometer under microscope.

### EdU incorporation assay

Cells were exposed to 10 µM of 5-ethynyl-2′-deoxyuridine (EdU; Thermo Fisher Scientific) for 1 h at 37 °C. The cells were fixed in 4% paraformaldehyde, permeabilized with 0.5% Triton X-100, and reacted with Click Additive Solution (Beyotime, Shanghai, China) for 30 min in the dark. Nuclei were stained with Hoechst 33,342 (Beyotime). Proliferating cells were visualized under a fluorescence microscope.

### Colony formation assay

Cells were seeded in 6-well plates (600 cells/well). After culturing for 10–14 days, the generated colonies were fixed with methanol for 30 min. The colonies were then stained by crystal violet and counted.

### Apoptosis assay

Cells were harvested and labeled with annexin V-fluorescein isothiocyanate (FITC) and propidium iodide (PI) as per the manufacturer’s instructions (Beyotime). The stained cells were analyzed with a FACSCalibur flow cytometer (Becton Dickinson, San Jose, CA, USA).

### Assessment of cisplatin sensitivity

Cells were seeded in 96-well plates and treated with indicated concentrations of cisplatin (Sigma-Aldrich) for 72 h. Cell viability was measured using 3-(4,5-dimethylthiazol-2-yl)-2,5-diphenyltetrazolium bromide (MTT; Sigma-Aldrich). Absorbance at 595 nm was recorded. The half maximal inhibitory concentration (IC_50_) of cisplatin was then determined.

### Tumorigenicity in nude mice

Male BALB/c nude mice (4–6 weeks old) were divided to 3 groups (*n* = 4 for each group). A total of 2 × 10^6^ A549 cells transfected with indicated plasmids were injected subcutaneously into nude mice. Tumor volume was measured every 5 days. Tumor growth curves were plotted. Twenty-five days later, the mice were sacrificed via CO_2_ inhalation. For assessment of the anticancer efficacy of anti-TM4SF1 in vivo, intratumoral injection of anti-TM4SF1 (catalog number: MABC1723; Sigma-Aldrich; 2 mg/kg body weight) was performed every 5 days after the tumors reaching a volume of approximately 150 mm^3^.

### Immunoprecipitation and mass spectrometry

For Flag-PLAU purification, cells were lysed in RIPA buffer with the protease inhibitor cocktail (Roche) and incubated with anti-FLAG M2 mAb (F3165; Sigma-Aldrich) or control IgG overnight. Flag-PLAU was eluted from beads with 100 µg/mL 3XFlag peptide (Sigma-Aldrich). For immunoprecipitation of endogenous PLAU, *ARID1A*-depleted A549 and H1299 cells were lysed and incubated with anti-PLAU (#15,800; Cell Signaling Technology) or IgG. The PLAU immunoprecipitates were captured by protein A/G agarose beads at 4 °C for 2 h and subjected to Western blot analysis.

For mass spectrometry analysis, Flag immunoprecipitates from *ARID1A*-depleted A549 cells were resolved by SDS-PAGE and analyzed by silver staining. Bands of silver stained-gels were cut, destained. and digested using 10 ng/µL of trypsin (Sigma-Aldrich). The resulting peptides were dried and resuspended in 30% acetonitrile and 0.1% trifluoroacetic acid before liquid chromatography-tandem mass spectrometry analysis. Mass spectra were processed using Proteome Discoverer 2.1 (Thermo Fisher Scientific). Proteins were identified by searching against UniProt database (*Homo sapiens*).

### Cycloheximide (CHX) chase assay

CHX chase assay was performed as described previously [25]. In brief, PLAU-overexpressing or control NSCLC cells were treated with 50 µg/mL CHX (Sigma-Aldrich), an inhibitor of protein biosynthesis, for 0–4 h and lysed for measurement of TM4SF1 protein levels by Western blot analysis.

### Statistical analysis

Data are expressed as the mean ± standard deviation from three independent experiments unless otherwise stated. Statistical analyses were performed using the Student’s *t*-test or one-way analysis of variance. *P*-values < 0.05 were considered statistically significant.

## Results

### *PLAU* is upregulated upon *ARID1A* loss

**Fig. 1:**
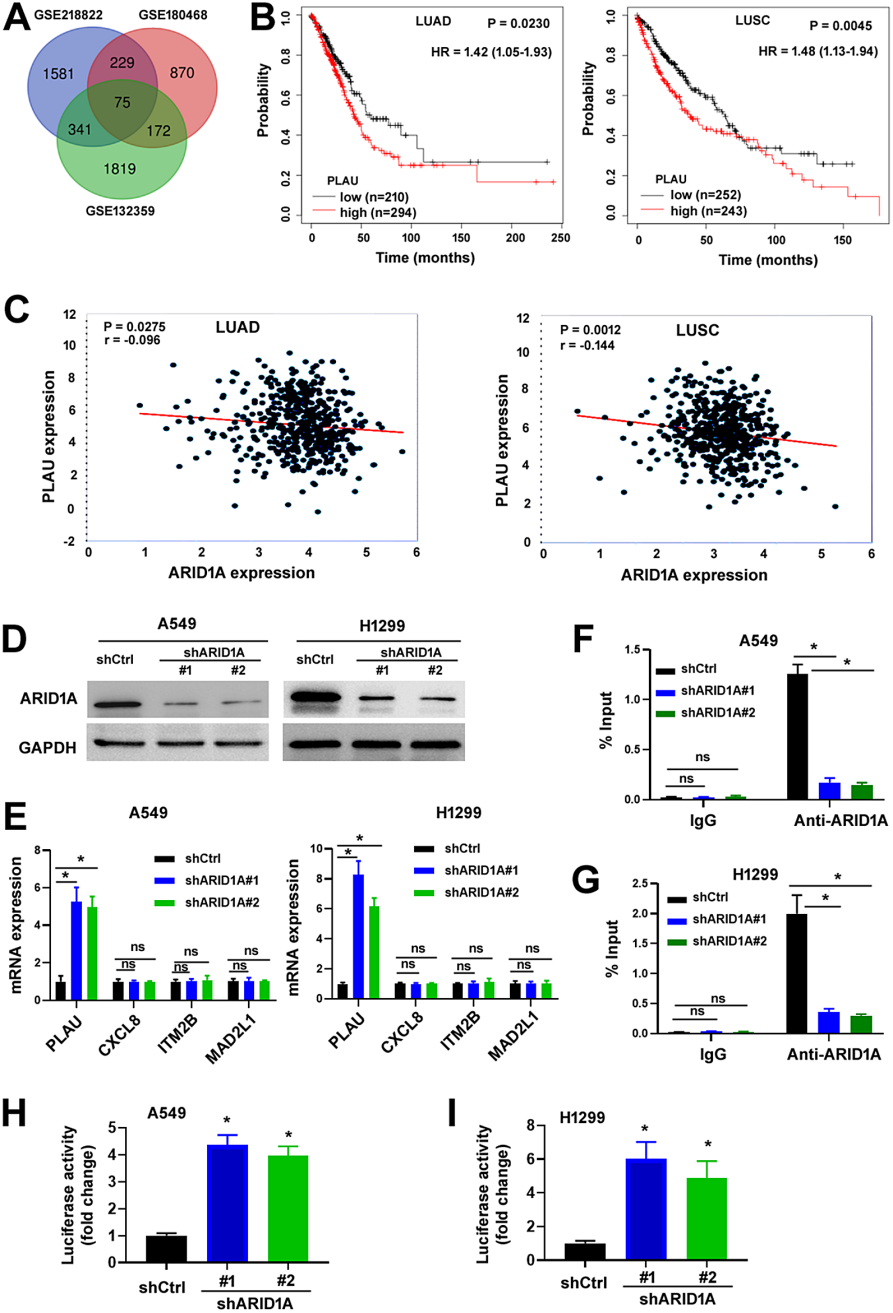
Upregulation of PLAU in ARID1A-deficient NSCLC cells. (**A**) Venn diagram of differentially expressed genes in GSE218822, GSE180468, and GSE132359. (**B**) Overall survival curves comparing the high and low expression of PLAU in lung adenocarcinoma (LUAD) and lung squamous cell carcinoma (LUSC) from Kaplan-Meier plotter. HR: hazard ratio. (**C**) Negative correlation between *PLAU* and *ARID1A* in LUAD and LUSC based on the ENCORI database. (**D**) Relative ARID1A protein levels determined by Western blot analysis in NSCLC cells transfected with 2 shRNAs targeting *ARID1A*. (**E**) Effects of 2 shRNAs targeting *ARID1A* on the expression of indicated genes. ^*^P < 0.05. ns indicates no significance. (F,G) ChIP assay. Chromatin from A549 (**F**) and H1299 (**G**) cells transfected with indicated shRNAs was immunoprecipitated with anti-ARID1A antibody or control IgG. DNA fragments of the promoter of *PLAU* were quantitated by real-time PCR analysis. (**H**, **I**) Measurement of the *PLAU* promoter-driven luciferase activities in A549 (**H**) and H1299 (**I**) cells transfected with indicated shRNAs. ^*^P < 0.05 relative to the shCtrl group

To identify novel target genes of *ARID1A* in malignant diseases, we analyzed 3 GEO datasets that were generated by RNA-sequencing using *ARID1A*-deficient and control cancer cells: GSE218822 in pancreatic cancer cells, GSE180468 in ovarian cancer cells, and GSE132359 in esophageal squamous cell cancer cells. A large number of DEGs were detected between *ARID1A*-deficient and control cancer cells. Venn diagram showed 75 DEGs overlapping among the 3 GEO datasets (Fig. 1A), suggesting their link with malignant progression. Overall survival analysis using the Kaplan-Meier plotter database demonstrated that 20 of the 75 common DEGs had potential prognostic significance in both LUAD and LUSC (Fig. 1B and Supplementary Figure S1). Co-expression analysis was performed for ARID1A and the 20 candidate genes using the ENCORI database. The results showed that *ARID1A* mRNA expression was negatively correlated with *CXCL8*, *ITM2B*, *MAD2L1* and *PLAU* and positively correlated with *KIAA1217*, *MICAL2* and *PELI1* in both LUAD and LUSC (Fig. 1C and Supplementary Figure S2). Given the ability of ARID1A to repress target gene transcription, here we focused on *CXCL8*, *ITM2B*, *MAD2L1* and *PLAU*. We found that *ARID1A* knockdown induced the expression of PLAU in A549 and H1299 cells, without altering the expression of *CXCL8*, *ITM2B*, or *MAD2L1* (Fig. 1D and E). Most importantly, ChIP assays indicated that *ARID1A* knockdown reduced the occupancy of the *PLAU* promoter by ARID1A protein (Fig. 1F and G). Luciferase reporter assays demonstrated that the *PLAU* promoter-driven luciferase activities were enhanced in ARID1A-depleted NSCLC cells, compared with the control cells (Fig. 1H and I). These data suggest that ARID1A negatively regulates the expression of PLAU in NSCLC cells via direct binding to the promoter of *PLAU*.

### PLAU promotes NSCLC growth, survival, and chemoresistance

**Fig. 2:**
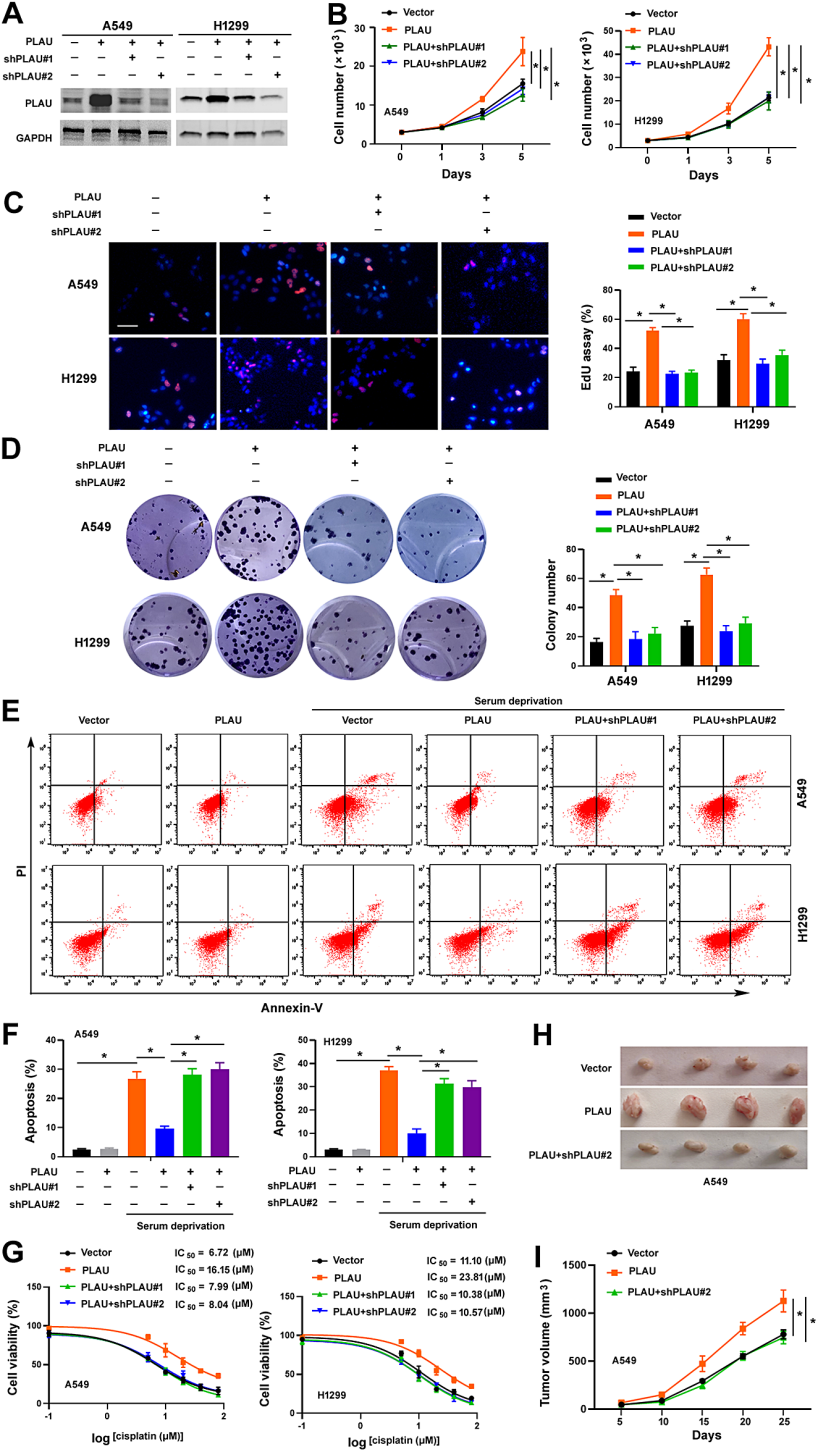
PLAU contributes to NSCLC growth, survival, and chemoresistance. (**A**) Western blot analysis of PLAU protein levels in A549 and H1299 cells transfected with indicated plasmids. (**B**) Assessment of the proliferation of NSCLC cells transfected with indicated plasmids by direct counting. ^*^P < 0.05. (**C**) NSCLC cell proliferation as measured by EdU assay. Left, representative images of EdU (red) and Hoechst 33,342 (blue) staining of the cells; scale bar = 50 μm. Right, quantification of EdU-positive cells. ^*^P < 0.05. (**D**) Colony formation assay. Left, representative wells showing colonies formed. Right, quantification of colony numbers. ^*^P < 0.05. (**E**, **F**) Apoptosis analysis by annexin-V and propidium iodide staining. NSCLC cells transfected with indicated plasmids were cultured in the presence or absence of serum for 48 h before apoptosis assay. ^*^P < 0.05. (**G**) NSCLC cells transfeced with indicated plasmids were treated with different concentrations of cisplatin for 72 h before viability assessment by the MTT method. (**H**, **I**) Tumorigenic studies. (**H**) Pictures of 4 representative tumors grown from A549 cells transfected with indicated plasmids. (**I**) Tumor volumes were calculated at indicated time points. ^*^P < 0.05

To explore the role of PLAU in NSCLC development and progression, we overexpressed PLAU in A549 and H1299 cells where endogenous PLAU was expressed at a low level (Fig. 2A). The growth rate of PLAU-overexpressing NSCLC cells was significantly increased compared to control cells (Fig. 2B and C). Consistently, forced expression of PLAU promoted colony formation in NSCLC cells (Fig. 2D). Furthermore, overexpression of PLAU attenuated serum deprivation-induced apoptosis (Fig. 2E and F) and decreased cisplatin sensitivity (Fig. 2G) in A549 and H1299 cells. To confirm the oncogenic role of PLAU, we impaired PLAU overexpression in NSCLC cells by transfecting *PLAU*-targeting shRNAs (Fig. 2A). As expected, depletion of *PLAU* reversed the aggressive phenotype observed in PLAU-overexpressing NSCLC cells (Fig. 2B and G). We further examined the effect of PLAU overexpression on the tumorigenicity of NSCLC cells in vivo. We found that the presence of exogenous PLAU significantly increased the growth of A549 cell-derived xenograft tumors, which was reversed by delivery of *PLAU*-targeting shRNAs (Fig. 2H and I). Taken together, our results indicate the oncogenic role of PLAU in NSCLC.

### The interaction with PLAU increases the stability of TM4SF1

**Fig. 3:**
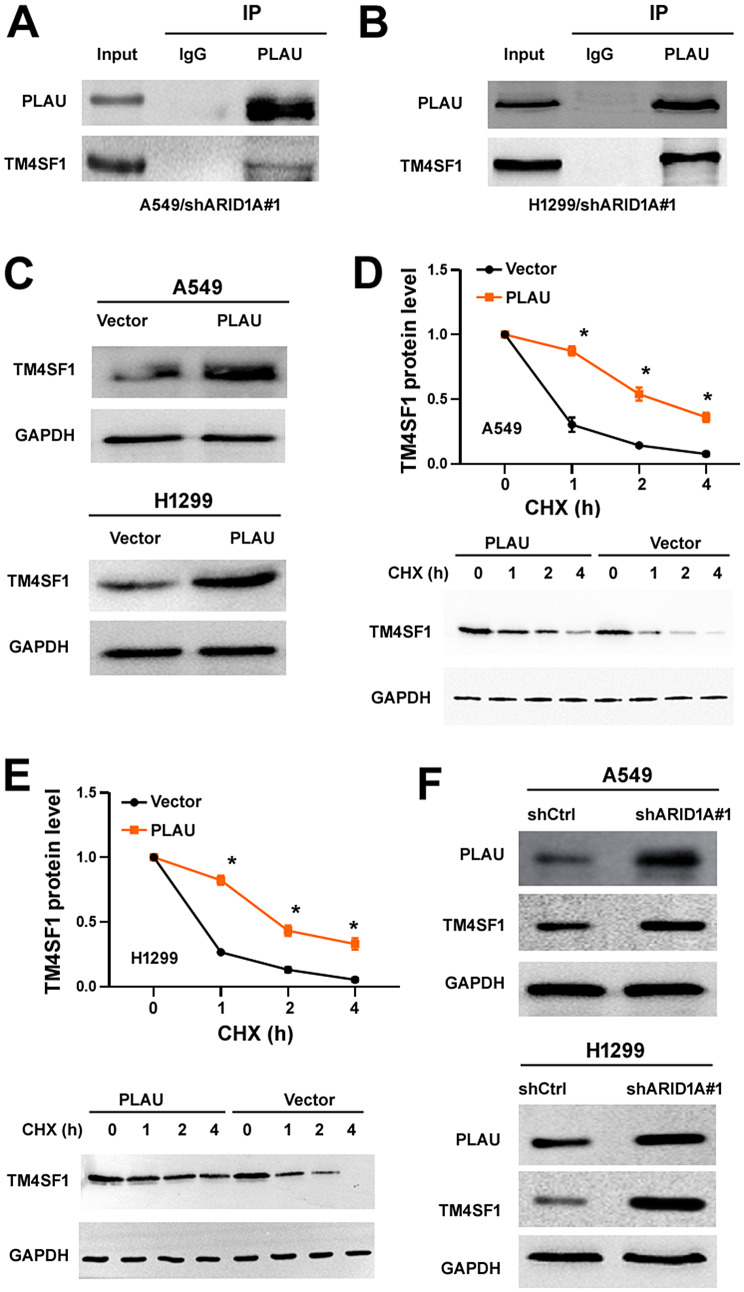
The interaction with PLAU increases the stability of TM4SF1. (**A**, **B**) Co-immunoprecipitation assays using anti-PLAU antibody in ARID1A-depleted A549 (**A**) and H1299 (**B**) cells. (**C**) Western blot analysis of TM4SF1 protein levels in A549 and H1299 cells transfected with indicated plasmids. (**D**, **E**) NSCLC cells were transfected with vector or PLAU-expressing plasmid and treated with CHX for the indicated time intervals. TM4SF1 protein levels were quantified by Western blot analysis. ^*^P < 0.05. (**F**) NSCLC cells were transfected with indicated shRNAs and tested for PLAU and TM4SF1 protein levels by Western blot analysis

Next, we sought to determine how PLAU induces aggressive phenotype in NSCLC cells. To this end, we searched for the key partner of PLAU in NSCLC cells by overexpressing Flag-tagged PLAU and performing mass spectrometry analysis of Flag immunoprecipitates. Many PLAU-interacting proteins were detected (Supplementary Table S2). Among them, we focused on one top candidate protein, TM4SF1, because it plays an oncogenic role in several malignant diseases including NSCLC [26–28]. We validated the interaction between endogenous PLAU and TM4SF1 in *ARID1A*-depleted A549 and H1299 cells by co-immunoprecipitation assays (Fig. 3A and B). Next, we assessed the regulation of TM4SF1 by PLAU. When PLAU was overexpressed in NSCLC cells, TM4SF1 protein but not mRNA levels were markedly increased (Fig. 3C and Supplementary Figure S3). CHX chase analysis of protein degradation revealed that overexpression of PLAU prevented the degradation of TM4SF1 (Fig. 3D and E). In *ARID1A*-depleted NSCLC cells, TM4SF1 protein levels were elevated along with PLAU protein levels (Fig. 3F). Our results collectively suggest that PLAU interacts with TM4SF1 protein to enhance its stability, thus contributing to the increase in the TM4SF1 protein level.

### TM4SF1 is required for PLAU-induced aggressive phenotype

**Fig. 4:**
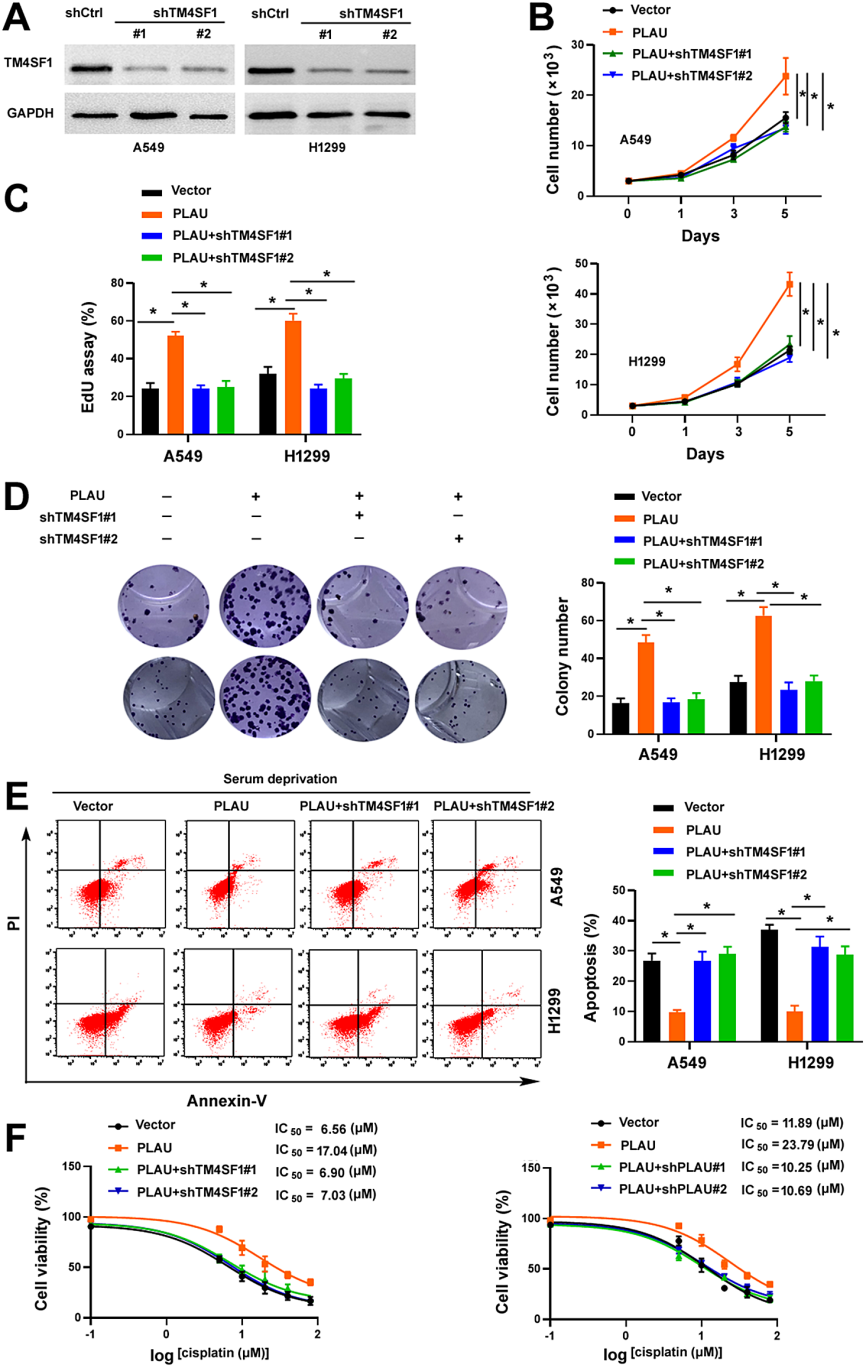
TM4SF1 is required for PLAU-induced aggressive phenotype. (**A**) Western blot analysis of TM4SF1 protein levels in A549 and H1299 cells transfected with shRNAs targeting *TM4SF1*. (**B**) Assessment of the proliferation of NSCLC cells transfected with indicated plasmids by direct counting. ^*^P < 0.05. (**C**) NSCLC cell proliferation as measured by EdU assay. ^*^P < 0.05. (**D**) Colony formation assay. ^*^P < 0.05. (**E**) Apoptosis analysis by annexin-V and propidium iodide staining. NSCLC cells transfected with indicated plasmids were cultured in the presence or absence of serum for 48 h before apoptosis assay. ^*^P < 0.05. (**F**) NSCLC cells transfeced with indicated plasmids were treated with different concentrations of cisplatin for 72 h before viability assessment by the MTT method

**Fig. 5:**
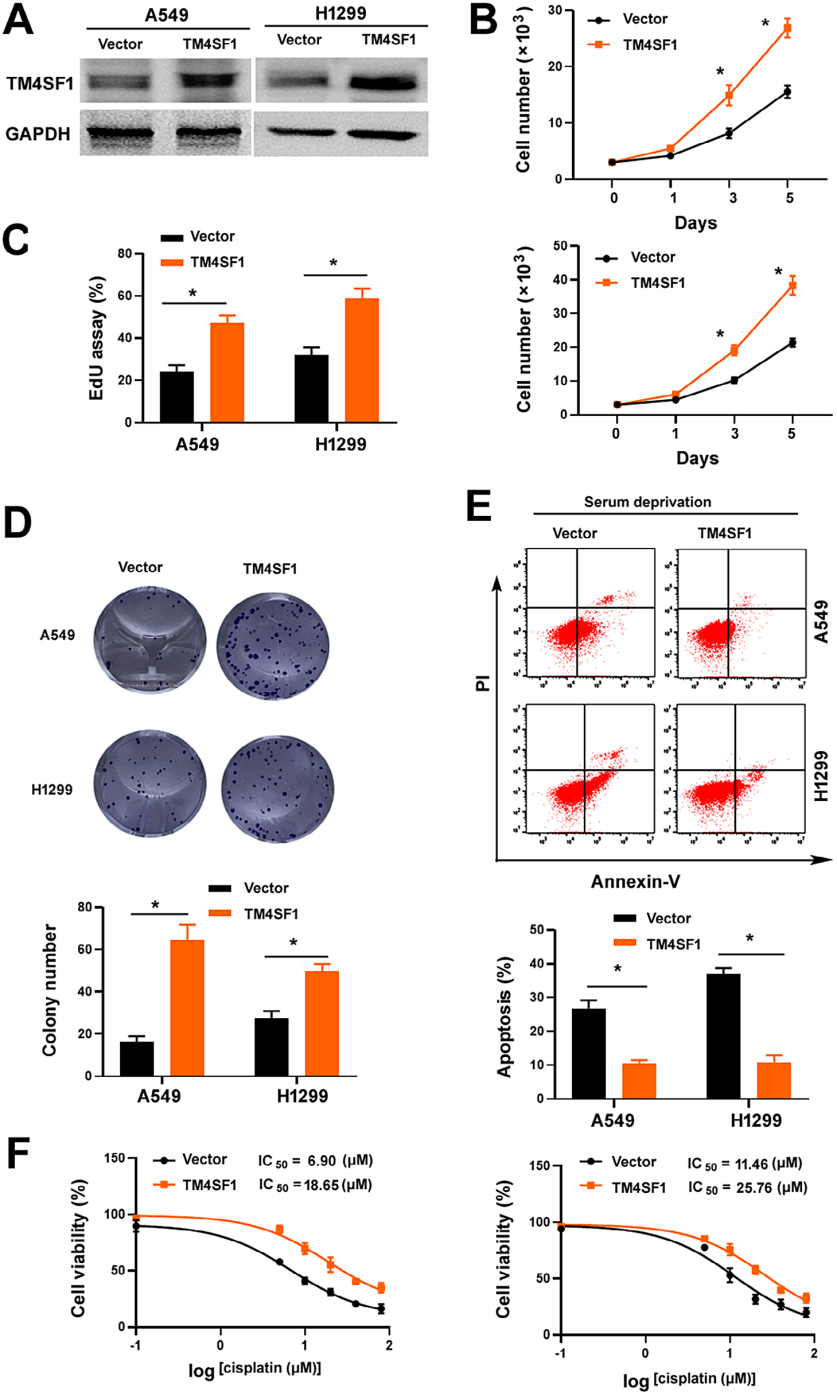
Overexpression of TM4SF1 promotes NSCLC cell proliferation and survival. (**A**) Western blot analysis of TM4SF1 protein levels in vector- and TM4SF1-overexpressing cells. GAPDH was used as a loading control. (**B**) Assessment of cell proliferation by direct counting. ^*^P < 0.05. (**C**) Cell proliferation as measured by EdU assay. ^*^P < 0.05. (**D**) Colony formation assay. ^*^P < 0.05. (**E**) NSCLC cells transfected with indicated plasmids were tested for apoptosis after culturing in the presence or absence of serum for 48 h. ^*^P < 0.05. (**F**) NSCLC cells transfeced with indicated plasmids were treated with different concentrations of cisplatin for 72 h before viability assessment by the MTT method

Next, we determined whether PLAU-induced aggressive phenotype in NSCLC cells depends on TM4SF1-mediated signaling. Knockdown of *TM4SF1* blocked the proliferation and colony formation of PLAU-overexpressing A549 and H1299 cells (Fig. 4A and D). Moreover, depletion of *TM4SF1* increased the apoptosis upon serum deprivation (Fig. 4E) and restored the sensitivity to cisplatin (Fig. 4F) in PLAU-overexpressing NSCLC cells. Overexpression of TM4SF1 led to similar phenotypes on A549 and H1299 cells, as did PLAU overexpression (Fig. 2). Specially, overexpression of TM4SF1 promoted NSCLC cell growth and rendered NSCLC cell more resistant to nutrient stress and cisplatin (Fig. 5A and F). Collectively, these data suggest that PLAU supports NSCLC cell growth and survival via a mechanism involving the interaction with TM4SF1.

### PLAU and TM4SF1 interaction leads to activation of Akt signaling to promote NSCLC cell growth and survival

**Fig. 6:**
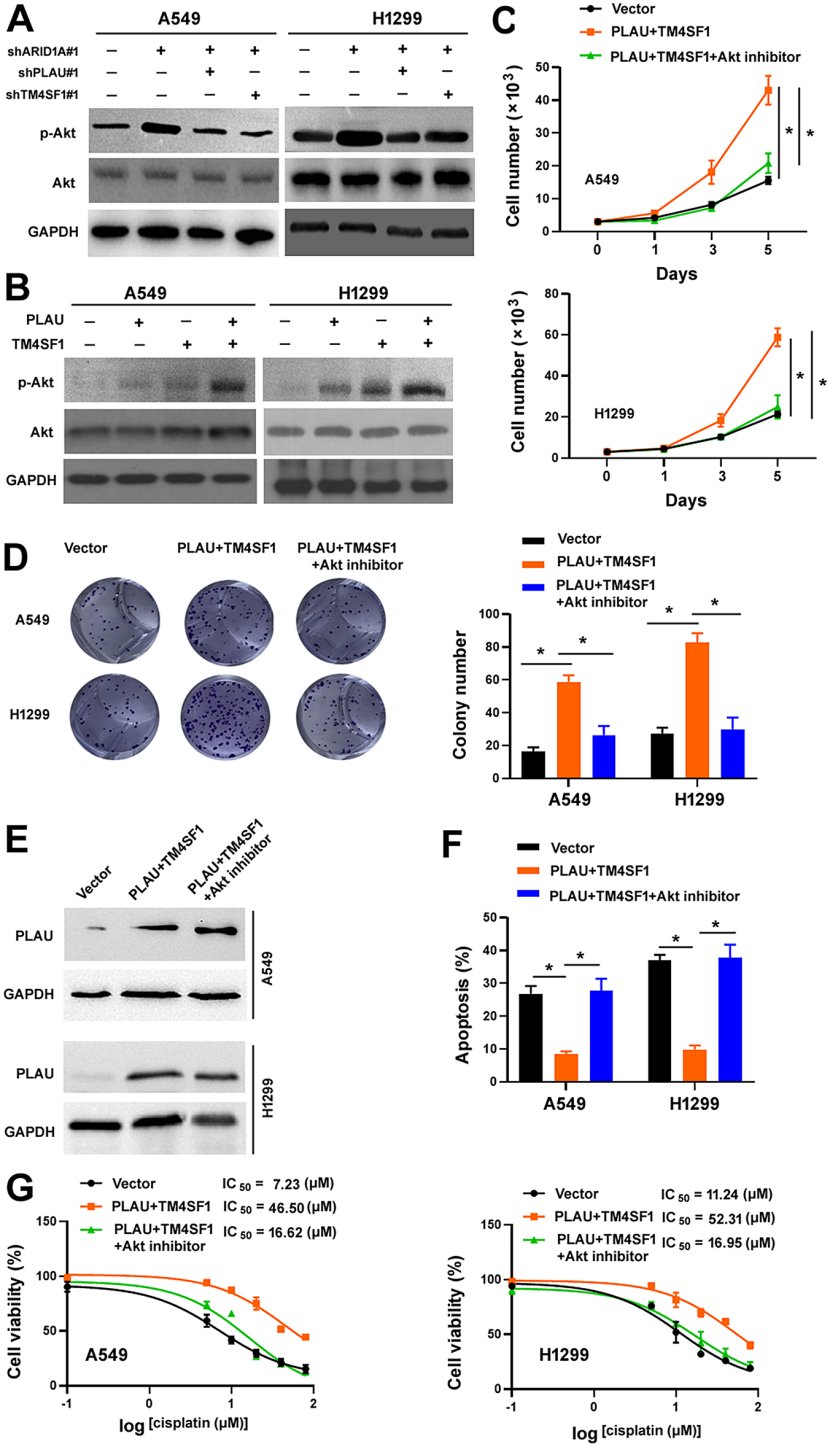
PLAU and TM4SF1 interaction leads to activation of Akt signaling to promote NSCLC cell growth and survival. (**A**, **B**) Western blot analysis of phosphorylated Akt (p-Akt) and total Akt in NSCLC cells transfected with indicated plasmids. (**C**) NSCLC cells were co-transfected with PLAU and TM4SF1 and treated with the Akt inhibitor MK2206. Cell proliferation was determined by cell counting. ^*^P < 0.05. (**D**) Colony formation assay in NSCLC cells co-transfected with PLAU and TM4SF1 and treated with the Akt inhibitor MK2206. ^*^P < 0.05. (**E**) Western blot analysis of PLAU protein expression in NSCLCs with indicated treatments. (**F**) NSCLC cells were co-transfected with PLAU and TM4SF1 and treated with the Akt inhibitor MK2206. Cell apoptosis was measured after culturing in the presence or absence of serum for 48 h. ^*^P < 0.05. (**G**) NSCLC cells were co-transfected with PLAU and TM4SF1 and treated with the Akt inhibitor MK2206. The cells were tested for viability after exposure to different concentrations of cisplatin for 72 h

Since TM4SF1 has the ability to regulate Akt signaling [26] and *ARID1A* loss leads to the activation of Akt signaling in lung cancer cells [14], we thus examined whether PLAU and TM4SF1 interaction is involved in the activation of Akt signaling in ARID1A-deficient lung cancer cells. We found that *ARID1A* depletion-induced activation (phosphorylation) of Akt was abolished when *PLAU* or *TM4SF1* was knocked down (Fig. 6A). Moreover, overexpression of PLAU and TM4SF1, alone or in combination, enhanced the activation of Akt in A549 and H1299 cells (Fig. 6B). When Akt activity was inhibited by a specific inhibitor, PLAU/TM4SF1 co-expression-induced NSCLC cell growth and survival was significantly suppressed (Fig. 6C and G). Inhibition of Akt did not alter the induction of PLAU protein expression upon overexpression of PLAU and TM4SF1 (Fig. 6E). Taken together, these results indicate that PLAU and TM4SF1 interaction promotes aggressive phenotype in ARID1A-deficient NSCLC cells through the activation of Akt signaling.

### Targeting TM4SF1 inhibits the growth and increases cisplatin cytotoxicity in *ARID1A* -depleted NSCLC cells

**Fig. 7:**
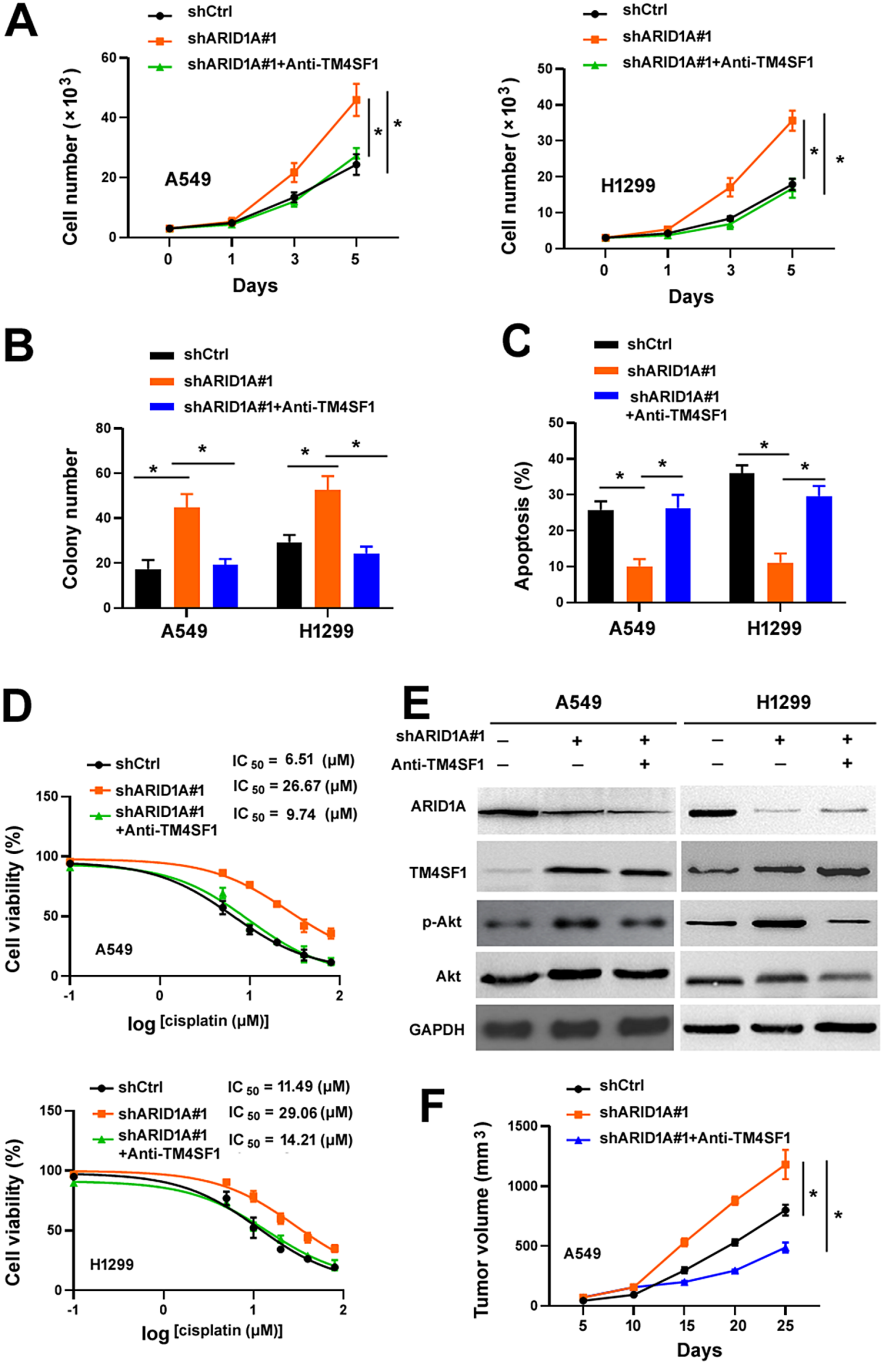
Targeting TM4SF1 inhibits the growth and increases cisplatin cytotoxicity in *ARID1A*-depleted NSCLC cells. (**A**) NSCLC cells were transfected with *ARID1A*-targeting shRNAs and treated with anti-TM4SF1 antibody. Cell proliferation was determined by direct counting. ^*^P < 0.05. (**B**) Colony formation assay in NSCLC cells transfected with *ARID1A*-targeting shRNAs and treated with anti-TM4SF1 antibody. ^*^P < 0.05. (**C**) NSCLC cells were transfected with *ARID1A*-targeting shRNAs and treated with anti-TM4SF1 antibody. Cell apoptosis was measured after culturing in the presence or absence of serum for 48 h. ^*^P < 0.05. (**D**) NSCLC cells were transfected with *ARID1A*-targeting shRNAs and treated with anti-TM4SF1 antibody. The cells were tested for viability after exposure to different concentrations of cisplatin for 72 h. (**E**) Western blot analysis of phosphorylated Akt (p-Akt) and total Akt in NSCLC cells after indicated treatments. (**F**) Effect of administration of anti-TM4SF1 antibody on the growth of ARID1A-depleted A549 xenograft tumors. ^*^P < 0.05

Next, we evaluated the therapeutic potential of targeting TM4SF1 in *ARID1A*-depleted NSCLC cells. Anti-TM4SF1 neutralizing antibody was used to block TM4SF1 activity. Of note, treatment with anti-TM4SF1 antibody suppressed cell growth and reduced cell survival in the presence of cisplatin (Fig. 7A and D). Western blot analysis confirmed that the addition of anti-TM4SF1 antibody decreased the phosphorylation of Akt in *ARID1A*-depleted NSCLC cells (Fig. 7E). In vivo studies further demonstrated that administration of anti-TM4SF1 antibody significantly abrogated the growth of *ARID1A*-depleted A549 xenograft tumors (Fig. 7F). These results suggest TM4SF1 as a therapeutic target for *ARID1A*-deficient NSCLC.

## Discussion

In the search for key genes in driving the progression and drug resistance of *ARID1A*-mutated NSCLC, we analyzed publicly available RNA-sequencing data acquired using different types of cancer cells with ARID1A ablation. The bioinformatic analysis reveals that PLAU is induced in ARID1A-depleted pancreatic cancer, ovarian cancer, and esophageal cancer cells. Consistently, our data show that PLAU expression is elevated in *ARID1A*-depleted NSCLC cells. ChIP assay further indicates the binding of ARID1A to the promoter of *PLAU*. ARID1A has the capacity to bind to DNA, which is essential for the promoter occupancy by the SWI/SNF complex [29]. The enrichment of ARID1A at the promoter of target genes leads to repression of gene transcription. Bitler et al. [30] reported that ARID1A directly binds to the promoter of *HDAC6* gene in ovarian cancer cells, consequently controlling *HDAC6* transcription. Similarly, in cholangiocarcinoma cells, ARID1A binding to the promoter of *ALDH1A1* blocks the transcription of *ALDH1A1* [31]. Our results, combined with these studies, suggest that ARID1A might repress the transcription of *PLAU* by directly binding to its promoter.

We have established an oncogenic role for PLAU in NSCLC. PLAU overexpression enhances NSCLC cell growth and colony formation. In vivo studies further demonstrate that overexpression of PLAU increases tumorigenicity of NSCLC cells. Our results are consistent with a previous study where PLAU overexpression promotes breast cancer cell growth [17]. Besides the regulation of cell growth, PLAU overexpression confers survival advantages to NSCLC cells. We show that PLAU-overexpressing NSCLC cells become less responsive to serum deprivation-induced apoptosis. Moreover, PLAU overexpression render NSCLC cells resistant to cisplatin. These results suggest that PLAU overexpression may represent a novel mechanism leading to chemoresistance in NSCLC cells. The link between PLAU and chemoresistance of cancer cells has also been observed in other cancer types [32, 33]. Inhibition of PLAU increases chemosensitivity of pancreatic cancer cells, which is associated with decreased stemness [32]. Cisplatin-resistant glioblastoma cells express higher levels of PLAU than parental control cells [33]. These findings prompt us to investigate the potential of PLAU as a biomarker for the chemosensitivity to cisplatin in NSCLC.

PLAU is known to exert its biological effects through interaction with a specific receptor, PLAUR [34]. The PLAU-PLAUR interaction regulates the proliferation, migration, and invasion of cancer cells [35, 36]. For instance, Ahmed et al. [35] reported that the PLAU/PLAUR binding induces Erk activation to promote the migration and invasion of colon cancer cells. However, our results suggest an alternative mechanism by which PLAU induces aggressive phenotype in NSCLC cells. Specially, we show that PLAU can interact with TM4SF1 and overexpression of PLAU increases the stability of TM4SF1. TM4SF1 is a small plasma membrane glycoprotein that functions as an oncogene [27]. Ye et al. [26] reported that TM4SF1 overexpression facilitates the proliferation, invasion, and chemoresistance in NSCLC cells. Our data show that knockdown of TM4SF1 impairs the aggressive phenotype in PLAU-overexpressing NSCLC cells, suggesting the dependence on TM4SF1. Moreover, ectopic expression of TM4SF1 phenocopies PLAU overexpression in NSCLC cells, increasing NSCLC cell growth and survival. These results support an important role for TM4SF1 in mediating PLAU oncogenic activity. Further studies demonstrate that the co-expression of PLAU and TM4SF1 leads to the activation of Akt signaling. It has been documented that activation of Akt signaling promotes lung adenocarcinoma growth and cisplatin resistance [37]. The Akt signaling pathway is involved in the progression of *ARID1A*-mutated NSCLC [14]. Notably, knockdown of PLAU or TM4SF1 blocks the activation of Akt induced by ARID1A deficiency. These results suggest that PLAU and TM4SF1 upregulation drives the activation of Akt signaling and defines the feature of *ARID1A*-mutated NSCLC.

TM4SF1 has been suggested as a promising anticancer target [38, 39]. Visintin et al. [39] reported that anti-TM4SF1 antibody conjugated with an auristatin cytotoxic agent shows anticancer activity in NSCLC, pancreatic, prostate, and colon cancers. Hence, we checked whether targeting TM4SF1 can restrain *ARID1A*-mutated lung cancer growth. Interestingly, blocking TM4SF1 with a specific antibody reduces the growth and increases cisplatin cytotoxicity in *ARID1A*-depleted NSCLC cells, which is coupled with inhibition of Akt activation. These results suggest that targeting TM4SF1 represents a therapeutic strategy for NSCLCs carrying *ARID1A* mutations.

## Conclusion

In conclusion, our data show that PLAU is induced in NSCLC cells with *ARID1A* loss and promotes NSCLC cell growth, survival, and cisplatin resistance through stabilization of TM4SF1. Disruption of the interaction between PLAU and TM4SF1 have potential therapeutic significance in the treatment of *ARID1A*-mutated NSCLC.

### Electronic supplementary material

Below is the link to the electronic supplementary material.

Supplementary Material 1

## Data Availability

The data that support the findings of this study are available from the corresponding author upon reasonable request.
