# Supplementary material for: PLAU promotes growth and attenuates cisplatin chemosensitivity in ARID1A-depleted non-small cell lung cancer through interaction with TM4SF1
Source: Biol Direct. 2024 Jan 17;19:7. doi: 10.1186/s13062-024-00452-7 (PMC10792809; doi:10.1186/s13062-024-00452-7)
Supplement: Supplementary file 1 — Supplementary Material 1 [file 13062_2024_452_MOESM1_ESM.doc]

**Supplementary data**

Supplementary Figure S1. Overall survival curves comparing the high and low expression of indicated genes in lung adenocarcinoma (LUAD) and lung squamous cell carcinoma (LUSC) from Kaplan-Meier plotter.


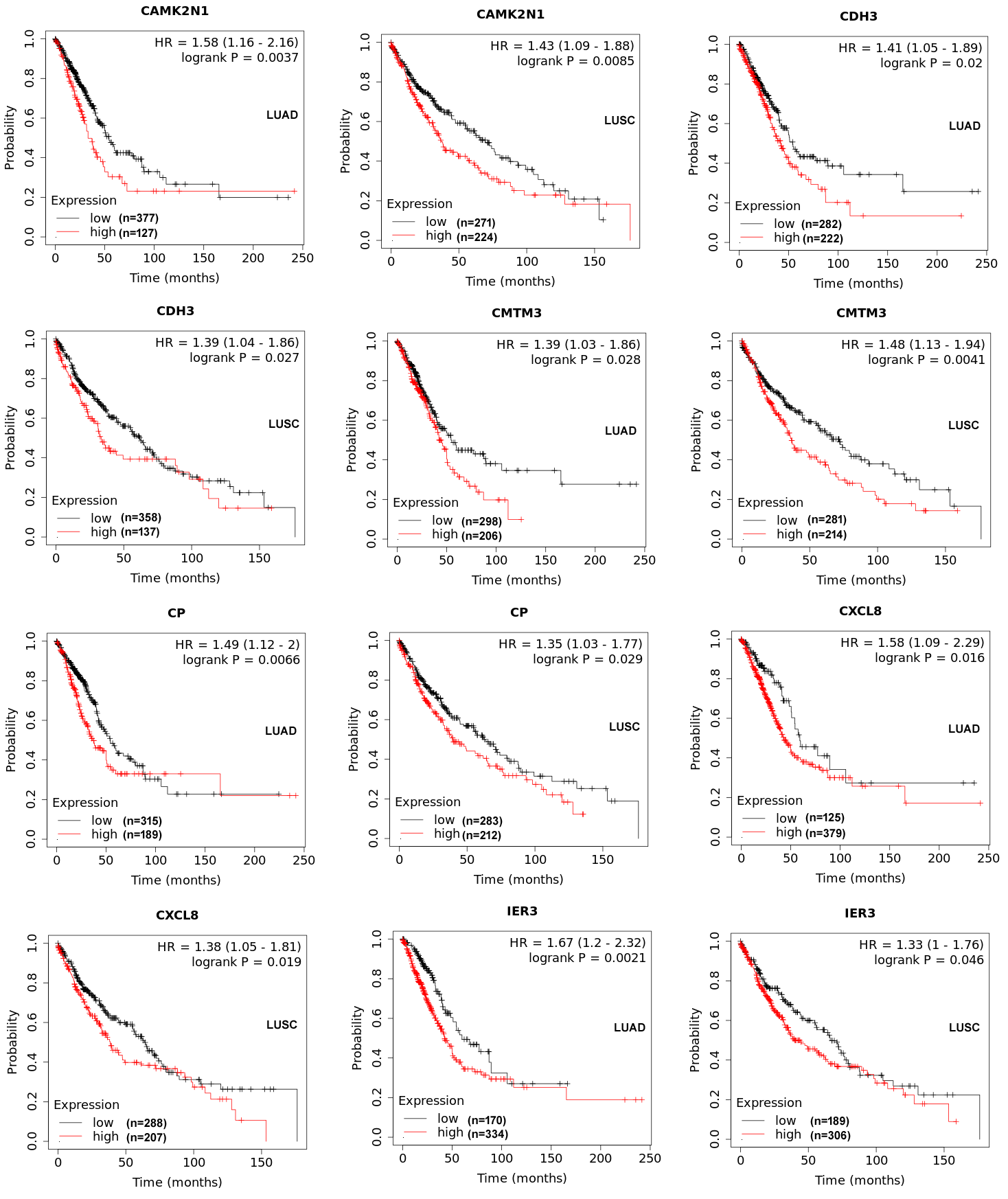


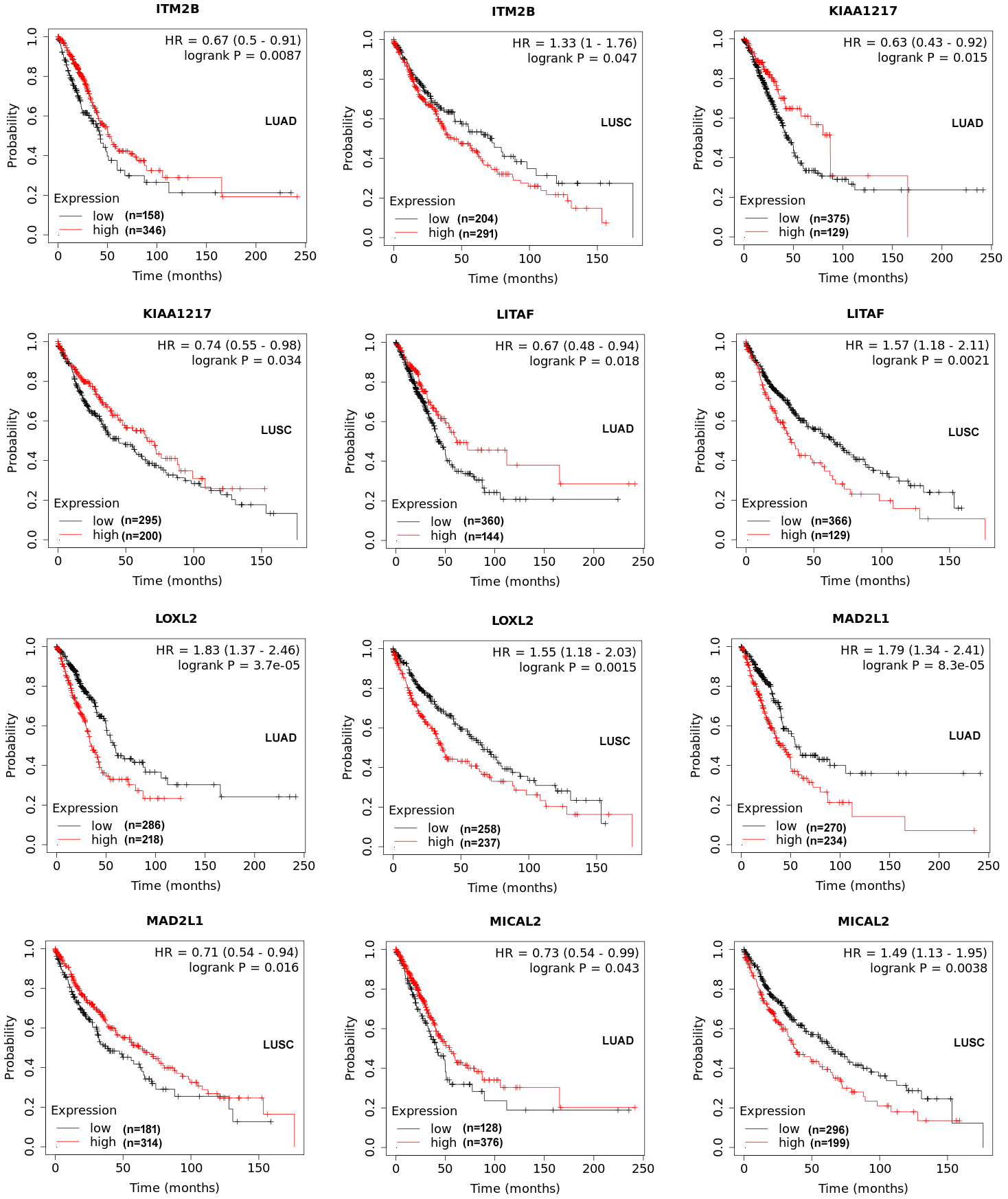


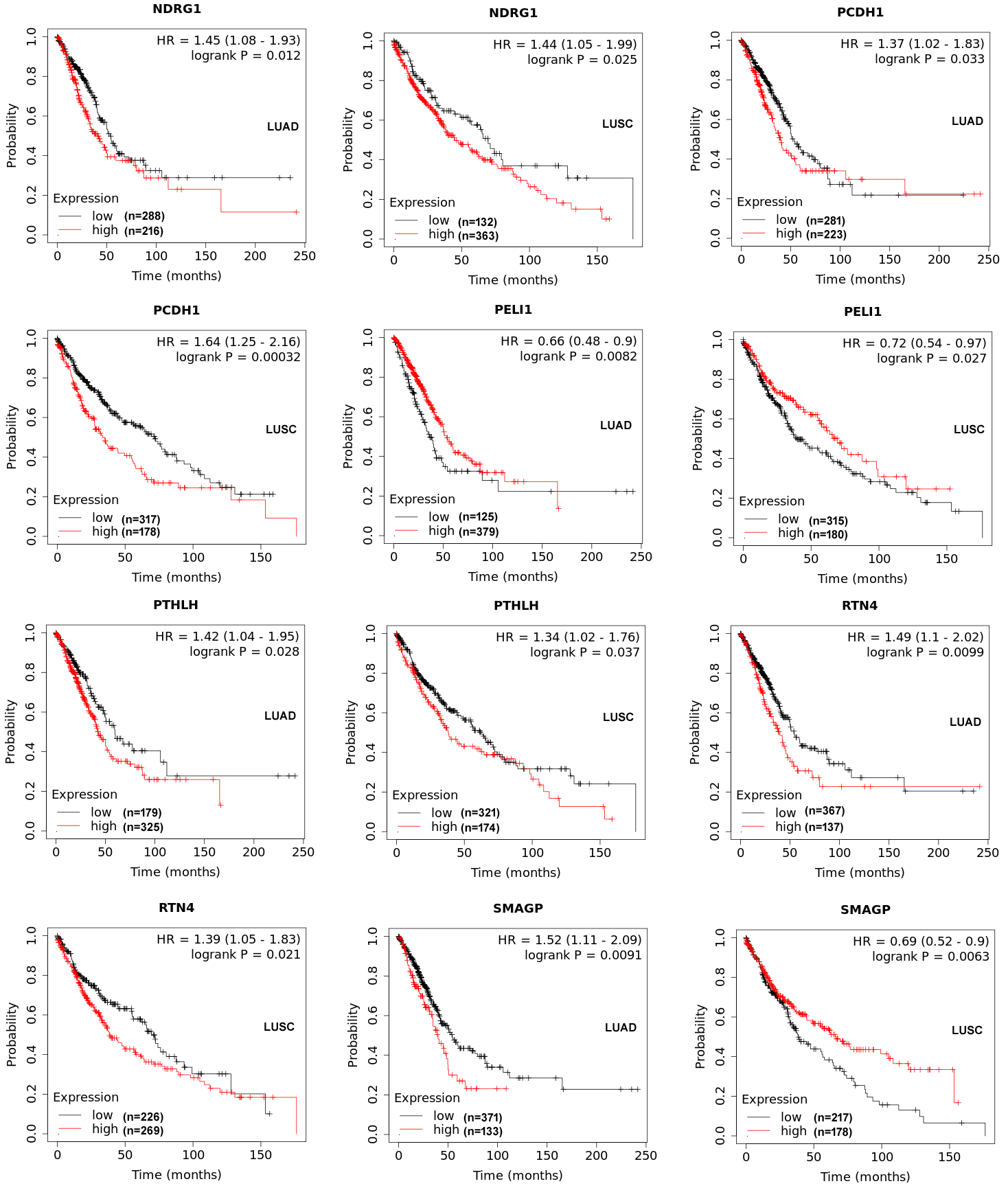


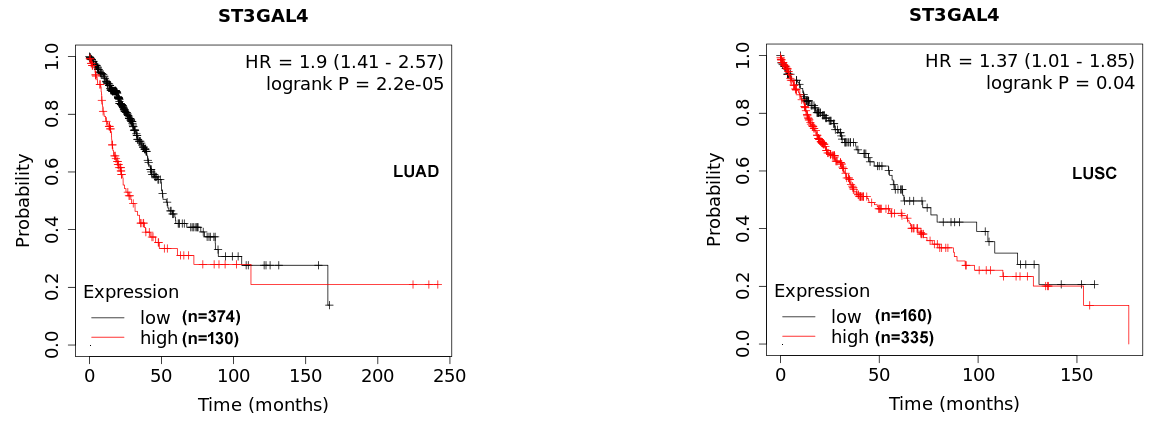


Supplementary Figure S2. Correlation between the genes of interest and *ARID1A* in LUAD and LUSC based on the ENCORI database.


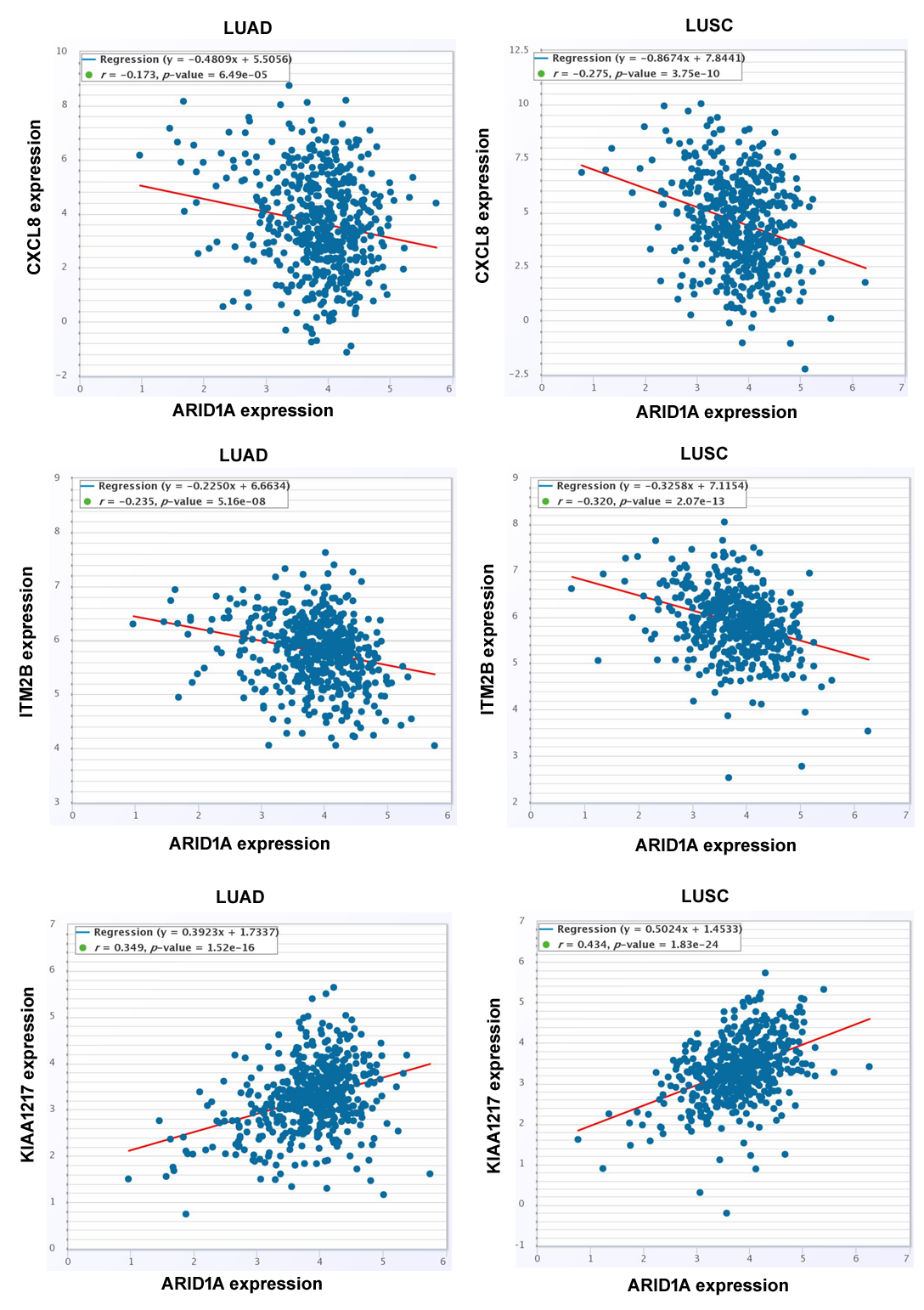


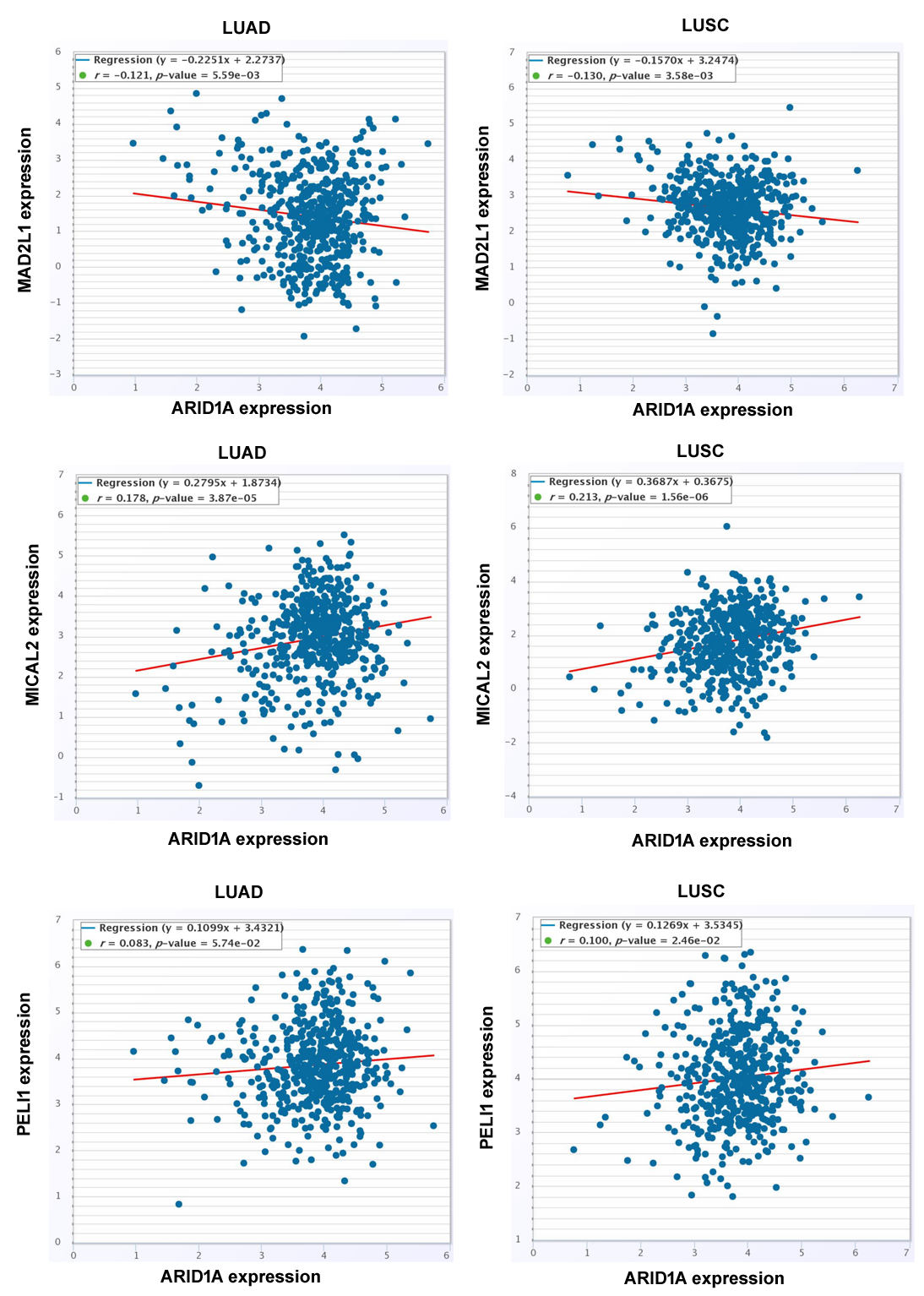


Supplementary Figure S3. Effect of PLAU overexpression on the expression of TM4SF1. ns indicates no significance.


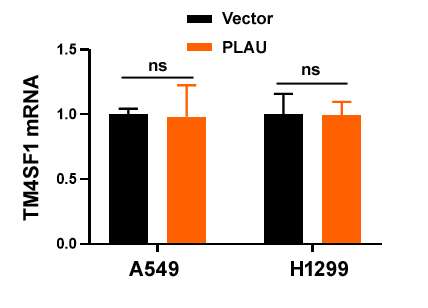


**Supplementary Table S1. Oligonucleotide sequences of primers and shRNAs**

| **Category** | **Name** | **Sequences** |
| --- | --- | --- |
| **shRNA** | shARID1A#1 | 5'-TAATGCCTTGCCCAATGCCAA-3' |
|  | shARID1A#2 | 5'-ACATGACCTATAATTATGCCA-3' |
|  | shPLAU#1 | 5'-CCATCAGCTGTAAGAAGAGAC-3' |
|  | shPLAU#2 | 5'-TTGCTGAAGATCCGTTCCAAG-3' |
|  | shTM4SF1#1 | 5'-AGGACAGAGCCACAATCTTCC-3' |
|  | shTM4SF1#2 | 5'-CCTCCACATGGTCCGAGTGCA-3' |
| **qRT-PCR** | CXCL8 forward | 5'-CACTGCGCCAACACAGAAAT-3' |
|  | CXCL8 reverse | 5'-CACCTCTTCAAAAACTTCTCCCG-3' |
|  | ITM2B forward | 5'-GCCTCAACCTTGAATGATGTTG-3' |
|  | ITM2B reverse | 5'-CTTGAGGTGAAATGAATCATAGG-3' |
|  | MAD2L1 forward | 5'-GTTCTTCTCATTCGGCATCAACA-3' |
|  | MAD2L1 reverse | 5'-GAGTCCGTATTTCTGCACTCG-3' |
|  | PLAU forward | 5'-GGACAAGCCAGGCGTCTACA-3' |
|  | PLAU reverse | 5'-AGAGCCTATCTTCCCAGTCTC-3' |
|  | GAPDH forward | 5'-TGCACCACCAACTGCTTAGC-3' |
|  | GAPDH reverse | 5'-GGCATGGACTGTGGTCATGAG-3' |
| **ChIP** | *PLAU* promoter (forward) | 5'-CTGTCTGTGCTTCTGGGAGA-3' |
|  | *PLAU* promoter (reverse) | 5'-GGTCCTCTAATCAGTTCTTCTG-3' |

**Supplementary Table S2. Mass spectrometry for proteins that bind to PLAU in *ARID1A*-depleted A549 cells**

| **Accession** | **Gene symbol** | **# Unique peptides** | **Spectrum counts** |
| --- | --- | --- | --- |
| P05783 | KRT18 | 8 | 118 |
| P42677 | RPS27 | 12 | 89 |
| P62241 | RPS8 | 30 | 85 |
| Q86UP2 | KTN1 | 9 | 76 |
| P60842 | EIF4A1 | 18 | 75 |
| P30408 | TM4SF1 | 14 | 50 |
| P39019 | RPS19 | 13 | 47 |
| P20020 | ATP2B1 | 10 | 42 |
| P46778 | RPL21 | 19 | 27 |
| P55060 | CSE1L | 9 | 24 |
| P15924 | DESP | 16 | 23 |
| P35527 | K1C9 | 7 | 22 |
| P09661 | SNRPA1 | 10 | 22 |
| P25786 | PSMA1 | 13 | 21 |
| P84090 | ERH | 10 | 18 |
| Q13501 | SQSTM1 | 8 | 16 |
| O00425 | IGF2BP3 | 5 | 16 |
| P29401 | TKT | 6 | 13 |
| P13645 | K1C10 | 7 | 13 |
| O00232 | PSMD12 | 4 | 12 |
| P35249 | RFC4 | 3 | 12 |
| P61247 | RS3A | 6 | 11 |
| O15020 | SPTN2 | 5 | 11 |
| Q86YZ3 | HORN | 5 | 10 |
| Q9UM54 | MYO6 | 2 | 10 |
| O75643 | U520 | 3 | 10 |
| Q15029 | U5S1 | 5 | 10 |
| Q9BW19 | KIFC1 | 6 | 9 |
| P60866 | RPS20 | 3 | 9 |
| P18621 | RPL17 | 3 | 8 |
| P23246 | SFPQ | 2 | 8 |
| P48681 | NEST | 3 | 8 |
| Q9Y3B7 | MRPL11 | 3 | 8 |
| O75223 | GGCT | 4 | 8 |
| Q9NS86 | LANCL2 | 2 | 8 |
| P61981 | YWHAG | 4 | 8 |
| Q5T3I0 | GPTC4 | 4 | 7 |
| Q14257 | RCN2 | 4 | 7 |
| O60306 | AQR | 5 | 7 |
| Q6PCB5 | RSBNL | 3 | 7 |
| O76094 | SRP72 | 2 | 7 |
| Q9BZJ0 | CRNL1 | 4 | 7 |
| Q8NBS9 | TXNDC5 | 3 | 7 |
| Q15542 | TAF5 | 4 | 7 |
| P07197 | NFM | 2 | 7 |
| Q06323 | PSME1 | 3 | 7 |
| Q16352 | AINX | 4 | 7 |
| R4GNH3 | PSMC3 | 3 | 7 |
| Q14684 | RRP1B | 5 | 7 |
| P21333 | FLNA | 4 | 6 |
| P08727 | K1C19 | 4 | 6 |
| P20700 | LMNB1 | 2 | 6 |
| Q99879 | H2BC14 | 3 | 6 |
| O95816 | BAG2 | 3 | 6 |
| P27635 | RL10 | 4 | 6 |
| P29966 | MARCKS | 2 | 6 |
| Q96N67 | DOCK7 | 3 | 6 |
| Q13162 | PRDX4 | 4 | 6 |
| P68366 | TBA4A | 4 | 6 |
| P23526 | AHCY | 3 | 6 |
| O00268 | TAF4 | 2 | 6 |
| P13647 | KRT5 | 4 | 6 |
| P38606 | ATP6V1A | 3 | 6 |
| P05198 | IF2A | 4 | 6 |
| Q14692 | BMS1 | 4 | 6 |
| Q96AB3 | ISOC2 | 2 | 6 |
| P26373 | RL13 | 3 | 6 |
| Q9H0A0 | NAT10 | 4 | 6 |
| Q13151 | HNRNPA0 | 4 | 6 |
| Q9BQG0 | MBB1A | 2 | 6 |
| P62269 | RS18 | 4 | 6 |
| P05388 | RPLP0 | 3 | 6 |
| P32969 | RPL9 | 4 | 6 |
| Q9H6F5 | CCD86 | 3 | 5 |
| Q9UMS4 | PRP19 | 2 | 5 |
| O43707 | ACTN4 | 3 | 5 |
| P19474 | RO52 | 3 | 5 |
| Q13435 | SF3B2 | 3 | 5 |
| Q9H0U4 | RAB1B | 3 | 5 |
| P49411 | EFTU | 3 | 5 |
| P81605 | DCD | 2 | 5 |
| P0DP25 | CALM3 | 3 | 5 |
| Q96DI7 | SNR40 | 4 | 5 |
| Q9HCS7 | SYF1 | 2 | 5 |
| P21675 | TAF1 | 3 | 5 |
| P53999 | TCP4 | 4 | 5 |
| P46781 | RS9 | 3 | 5 |
| P23396 | RS3 | 4 | 5 |
| O60508 | PRP17 | 3 | 5 |
| Q9Y230 | RUVB2 | 3 | 5 |
| P07437 | TBB5 | 4 | 5 |
| Q96ME7 | ZN512 | 3 | 5 |
| Q9NVP1 | DDX18 | 4 | 5 |
| P04843 | RPN1 | 4 | 5 |
| P22531 | SPR2E | 4 | 5 |
| P25789 | PSMA4 | 4 | 5 |
| P07954 | FH | 3 | 5 |
| P24534 | EEF1B2 | 4 | 5 |
| O14744 | ANM5 | 4 | 5 |
| Q01469 | FABP5 | 5 | 5 |
| Q9Y265 | RUVB1 | 4 | 5 |
| P61158 | ARP3 | 4 | 4 |
| Q6YN16 | HSDL2 | 3 | 4 |
| P61019 | RAB2A | 3 | 4 |
| Q96GM5 | SMARCD1 | 2 | 4 |
| Q15361 | TTF1 | 3 | 4 |
| P22087 | FBRL | 2 | 4 |
| P09651 | ROA1 | 2 | 4 |
| P35232 | PHB | 2 | 4 |
| Q96A23 | CPNE4 | 4 | 4 |
| Q93084 | ATP2A3 | 1 | 4 |
| O94776 | MTA2 | 2 | 4 |
| Q02539 | H11 | 2 | 4 |
| P62979 | RS27A | 3 | 4 |
| O43491 | E41L2 | 3 | 4 |
| Q9Y4Z0 | LSM4 | 1 | 4 |
| Q8N3J9 | ZN664 | 2 | 4 |
| Q8WVS4 | DC2I1 | 4 | 4 |
| Q6P1X5 | TAF2 | 3 | 4 |
| P06396 | GELS | 3 | 4 |
| Q4VC05 | BCL7A | 3 | 4 |
| Q6ZRV2 | FA83H | 2 | 4 |
| Q8NFH3 | NUP43 | 3 | 4 |
| Q9Y6A4 | CFA20 | 4 | 4 |
| O60783 | RT14 | 3 | 4 |
| Q14247 | SRC8 | 2 | 4 |
| P47756 | CAPZB | 4 | 4 |
| P36873 | PP1G | 2 | 4 |
| Q08554 | DSC1 | 2 | 4 |
| P46977 | STT3A | 1 | 4 |
| J3QQX2 | ARHGDIA | 4 | 4 |
| Q09161 | NCBP1 | 4 | 4 |
| Q9UDW1 | QCR9 | 3 | 4 |
| Q07955 | SRSF1 | 3 | 4 |
| P52907 | CAZA1 | 2 | 4 |
| P62841 | RS15 | 4 | 4 |
| Q9H6R4 | NOL6 | 4 | 4 |
| P60903 | S10AA | 2 | 3 |
| Q15814 | TBCC | 2 | 3 |
| Q9BVM4 | GGACT | 3 | 3 |
| O43251 | RBFOX2 | 2 | 3 |
| O75694 | NU155 | 2 | 3 |
| P22314 | UBA1 | 3 | 3 |
| P33240 | CSTF2 | 2 | 3 |
| P01876 | IGHA1 | 1 | 3 |
| P07237 | PDIA1 | 3 | 3 |
| P41219 | PERI | 2 | 3 |
| P07900 | HS90A | 2 | 3 |
| P60660 | MYL6 | 3 | 3 |
| Q9UHD8 | SEPTIN9 | 3 | 3 |
| Q9BQ67 | GRWD1 | 2 | 3 |
| Q9Y3U8 | RPL36 | 3 | 3 |
| Q9H9T3 | ELP3 | 2 | 3 |
| Q9UI12 | ATP6V1H | 3 | 3 |
| Q8NDT2 | RB15B | 2 | 3 |
| P01833 | PIGR | 3 | 3 |
| Q9BSD7 | NTPCR | 2 | 3 |
| P46776 | RL27A | 3 | 3 |
| O00571 | DDX3X | 3 | 3 |
| O43447 | PPIH | 2 | 3 |
| O15144 | ARPC2 | 3 | 3 |
| O60294 | LCMT2 | 2 | 3 |
| Q14964 | RAB39A | 2 | 3 |
| O14545 | TRAFD1 | 1 | 3 |
| Q13868 | EXOSC2 | 3 | 3 |
| Q6P4A8 | PLBL1 | 2 | 2 |
| O75489 | NDUFS3 | 2 | 2 |
| O60884 | DNJA2 | 2 | 2 |
| Q9H0E2 | TOLIP | 2 | 2 |
| O75683 | SURF6 | 2 | 2 |
| P55795 | HNRH2 | 2 | 2 |
| Q15058 | KIF14 | 2 | 2 |
| Q9Y224 | RTRAF | 2 | 2 |
| Q56VL3 | OCIAD2 | 2 | 2 |
| P59665 | DEF1 | 1 | 2 |
| Q9HAV4 | XPO5 | 2 | 2 |
| P08574 | CY1 | 2 | 2 |
| P07384 | CAN1 | 2 | 2 |
| Q96HS1 | PGAM5 | 2 | 2 |
| Q6PI98 | IN80C | 2 | 2 |
| Q8IYB3 | SRRM1 | 2 | 2 |
| Q15427 | SF3B4 | 2 | 2 |
| O14639 | ABLM1 | 2 | 2 |
| P48047 | ATP5PO | 2 | 2 |
| Q9NQ50 | RM40 | 2 | 2 |
| P82930 | RT34 | 1 | 2 |
| Q9GZR2 | REXO4 | 2 | 2 |
| Q9UPW5 | AGTPBP1 | 2 | 2 |
| P19013 | K2C4 | 2 | 2 |
| P62424 | RL7A | 2 | 2 |
| P18583 | SON | 1 | 2 |
| Q8IWS0 | PHF6 | 1 | 1 |
| Q92734 | TFG | 1 | 1 |
| Q13573 | SNW1 | 1 | 1 |
| Q6IA86 | ELP2 | 1 | 1 |
| Q16698 | DECR1 | 1 | 1 |
| Q02878 | RL6 | 1 | 1 |
| P62857 | RPS28 | 1 | 1 |
| O75934 | SPF27 | 1 | 1 |
| Q9NW64 | RBM22 | 1 | 1 |
| P00390 | GSR | 1 | 1 |
